# Targeted plasma eQTL and pQTL analyses to identify potential novel biomarkers for Alzheimer's Disease in African Americans

**DOI:** 10.1002/alz70856_104423

**Published:** 2025-12-25

**Authors:** Vanessa M. Colón Badillo, Joseph S. Reddy, Jiangli Jin, Sarah J. Lincoln, Charlotte C.G. Ho, Julia E. Crook, Xue Wang, Kimberly G. Malphrus, Thuy T. Nguyen, Lindsey A. Kuchenbecker, Jean M Marrero Polanco, Nicole Tamvaka, Christian Lachner, Gregory S Day, John A. Lucas, Neill R. Graff‐Radford, Nilüfer Ertekin‐Taner, Minerva M. Carrasquillo

**Affiliations:** ^1^ Mayo Clinic, Jacksonville, FL, USA; ^2^ Mayo Clinic in Florida, Jacksonville, FL, USA

## Abstract

**Background:**

African Americans (AA) are twice as likely to develop dementia than non‐Hispanic Whites but remain underrepresented in Alzheimer's Disease (AD) research. Recent studies have identified AA specific risk factors for AD. Therefore, targeted biomarkers and/or therapies may be needed to effectively treat AD in AA. In this study, we aimed to determine if genetic variants that show association with AD‐risk, plasma transcript or protein levels in AA, may serve as accurate and accessible AD biomarkers.

**Method:**

In 189 clinically diagnosed AA AD cases and 183 AA cognitively unimpaired (CU) controls, we performed targeted DNA sequencing of 10 AD‐associated loci. Genetic variants at these loci were tested for association with corresponding plasma transcripts and total tau protein levels previously measured in this cohort using a custom nanoString panel and Simoa assays, respectively. Utilizing phased haplotypes (SHAPEIT4) from target sequencing, we inferred local ancestry (RFMix v2) using five superpopulations from the 1000 Genomes Project as anchors. Subsequently, we tested the association of each ancestry specific allelic dosages with endophenotypes using Tractor. In receiver operating characteristic (ROC) analyses, the most significant e/pQTLs were added sequentially to a base model that included age and sex to identify e/pQTLs that improved the accuracy to correctly classify AD cases and CU controls.

**Result:**

Of the 5,112 variants that were tested, 70 were nominally associated with AD‐risk and with plasma transcript or protein levels. Seven of these 70 variants also showed nominally significant associations of AD‐risk and transcript levels with either African or East Asian local ancestry. ROC analysis of age, sex, 33 e/pQTLs in *APOE*, *SORL1*, *TLR4*, *EPHA1*, *ABCA7*, *CLU*, and *CR1*, and plasma levels of *APP, ABCA7, AKAP9, CD14, CLU, and APOE*‐ɛ4 dosage achieved 88.2% area under the curve to discriminate AD vs. CU, a 30.4% improvement over the base model that only included age and sex.

**Conclusion:**

Plasma transcript levels and e/pQTLs are promising diagnostic biomarkers for AD that may improve accessibility and reduce costs for a more accurate diagnosis of AD.

